# CircPan3 Promotes the Ghrelin System and Chondrocyte Autophagy by Sponging miR-667-5p During Rat Osteoarthritis Pathogenesis

**DOI:** 10.3389/fcell.2021.719898

**Published:** 2021-11-19

**Authors:** Jing Zeng, Zhenzhen Zhang, Qing Liao, Qijin Lu, Jiemei Liu, Lixia Yuan, Gang Liu

**Affiliations:** ^1^Department of Rehabilitation Medicine, The Third Affiliated Hospital of Southern Medical University, Southern Medical University, Guangzhou, China; ^2^Department of Rehabilitation Medicine, Nanfang University of Science and Technology Hospital, Shenzhen, China; ^3^Department of Rehabilitation Medicine, Hankou Hospital, Wuhan, China; ^4^Department of Rehabilitation Medicine, Shunde Hospital of Southern Medical University, Southern Medical University, Foshan, China; ^5^School of Traditional Chinese Medicine, Southern Medical University, Guangzhou, China; ^6^Department of Rehabilitation Medicine, Nanfang Hospital, Southern Medical University, Guangzhou, China

**Keywords:** circPAN3, miR-667-5p, ghrelin, chondrocyte autophagy, osteoarthritis

## Abstract

This study aimed to investigate the potential roles of circRNAs in regulating osteoarthritis (OA)-related ghrelin synthesis, autophagy induction, and the relevant molecular mechanisms. Results showed that Col2a1, Acan, ghrelin, and autophagy-related markers expression were downregulated, while matrix metalloproteinase 13 (MMP13) and a disintegrin and metalloproteinase with thrombospondin motifs 5 (ADAMTS5) expressions increased in both IL-1β-induced rat chondrocytes and cartilage tissues of OA rats. A total of 130 circRNAs and 731 mRNAs were differentially expressed in IL-1β-induced rat chondrocytes. Among them, we found that circPan3 expression was significantly decreased in both cellular and animal OA models. CircPan3 directly targeted miR-667-5p. CircPan3 overexpression promoted Col2a1, Acan, ghrelin, beclin 1, and LC3-II expression but reduced MMP13 and ADAMTS5 expression in rat chondrocytes, whereas overexpression of miR-667-5p exhibited opposite effects on the above markers. Furthermore, we found that miR-667-5p bound directly to the 3′-UTR sequence of ghrelin gene. Moreover, the circPan3-induced alterations in chondrocytes were antagonized by miR-667-5p overexpression. Taken together, our findings demonstrate that circPan3 promotes ghrelin synthesis and chondrocyte autophagy via targeting miR-667-5p, protecting against OA injury. This study provided experimental evidence that circPan3/miR-667-5p/ghrelin axis might serve as targets of drug development for the treatment of OA.

## Introduction

Osteoarthritis (OA) is a common chronic joint disease characterized by articular cartilage degeneration and subsequent destruction of cartilage and bone. OA usually affects weight-bearing joints, including the knees and hips (Lambova and Müller-Ladner, 2018; Yu and Zhao, 2019). Over recent decades, OA has become a leading cause of disability and pain worldwide, showing an occurrence rate of 10–20% among the population aged over 50 years (Blanco et al., 2018; Lambova and Müller-Ladner, 2018). Previous etiological investigations revealed that the pathogenesis of OA is closely associated with many risk factors, such as age, female sex, genetic predisposition, mechanical stress, obesity, and joint injury (Lambova and Müller-Ladner, 2018; Yu and Zhao, 2019). Under physiological conditions, the articular cartilage consists of extracellular matrix and the chondrocytes (Lambova and Müller-Ladner, 2018). The extracellular matrix mainly includes collagen type II (Col-II), Col-IX, Col-XI, and proteoglycans-mainly aggrecan (Acan), which are synthesized by chondrocytes and other cell types (Lambova and Müller-Ladner, 2018). The Col-II and Acan contents in the articular cartilages were significantly decreased mainly due to abnormal chondrocytes, while the secretion of cartilage-degrading metalloproteinases (MMP13 and ADAMTS5) was elevated (Xie et al., 2017; Lambova and Müller-Ladner, 2018). These abnormal catabolic processes are mainly induced by excessive generation of interleukin-1β (IL-1β) and tumor necrosis factor alpha (TNF-α). They inhibited the synthesis of the extracellular matrix components (e.g., Col-II and Acan) in chondrocytes (Qu et al., 2017; Lambova and Müller-Ladner, 2018). However, the molecular mechanisms underlying the development of OA remain poorly unknown.

Ghrelin (GHRL) is an orexigenic peptide hormone that is synthesized in the stomach. GHRL promotes food intake, leading to increased body weight through interacting with the growth hormone secretagogue receptor (GHSR) and regulating the secretion of the pituitary growth hormone (GH) (Tschöp et al., 2000; Mani and Zigman, 2017). Hence, the GHRL system has also been proposed as a promising target for obesity intervention (Schellekens et al., 2010; Mani and Zigman, 2017). Besides, the GHRL system is also produced in various organs and involved in several other physiological and pathogenic processes, including blood glucose metabolism, cardiac and gastrointestinal functions, endocrine and exocrine pancreatic secretion modulation, bone formation, inflammation inhibition, psychosocial stress, cachexia, and anorexia nervosa (Delporte, 2013; Mani and Zigman, 2017). More importantly, GHRL has also been considered as a protective agent against the incidence and development of OA in recent years (Zou et al., 2016; Liu et al., 2018; Qu et al., 2018). For instance, GHRL effectively repressed the production of inflammatory cytokines and chondrocyte apoptosis while elevated the expression of Col-II and Acan in chondrocytes by modulating protein kinase B (Akt) and nuclear factor-kappa B (NF-κB) signaling during OA development (Qu et al., 2018).

The development and progression of OA are associated with insufficient autophagy in chondrocytes (Zhang et al., 2015; Yu and Zhao, 2019). Autophagy activation inhibited the mammalian target of rapamycin (mTOR) and thus effectively attenuated OA lesion in a mouse OA model (Shen et al., 2019). Autophagy was shown to be regulated by the GHRL system (Xu et al., 2017; Zhu et al., 2017). GHRLregulated the expression of autophagy-related genes through Akt and other signaling pathways in the pathogenesis of obesity, heart failure, and other diseases (Yuan and Wang, 2020). However, the specific molecular mechanisms by which the ghrelin system regulates the autophagy process during OA pathogenesis is still unclear.

Non-coding RNAs have been identified as potent regulators of gene expression under various biological and pathogenic processes, including OA development (Miyaki and Asahara, 2012; Su et al., 2016; Hu et al., 2018; Yu and Zhao, 2019). For instance, several microRNAs (miRNAs), such as miR-140, were reported to regulate the expression of OA-related genes and mediate the chondrocyte alterations associated with OA pathogenesis (Miyaki and Asahara, 2012; Yu and Zhao, 2019). Specifically, miRNA-145 suppressed the TNF-α-induced cartilage matrix degradation during OA development by directly suppressing the expression of the mitogen-activated protein kinase kinase 4 (*MKK4*) gene (Hu et al., 2017). A set of miRNAs modulated OA development via regulating the chondrocyte autophagy process (Yu and Zhao, 2019). The expression of miR-335-5p was significantly downregulated in peripheral blood mononuclear cells (PBMCs) of osteoarthritis patients, suggesting that miR-335-5p might be a protective molecule for osteoarthritis (He et al., 2017). MiRNA-335-5p inhibited chondrocyte inflammation during OA development via activating autophagy (Zhong et al., 2019).

Circular RNAs (circRNAs), another group of non-coding RNAs, exert biological roles mainly by acting as miRNA sponges. circRNAs also perform essential roles in the regulation of chondrocytes and OA development (Li et al., 2017; Zhou et al., 2018, 2019; Shen et al., 2019). Circ-0045714 regulated the synthesis of extracellular matrix components and the proliferation and apoptosis of chondrocytes in OA by sponging miR-193b, which targets the insulin-like growth factor 1 receptor (*IGF1R*) gene (Li et al., 2017). The interaction between circRNAs and miRNAs attributing to the ghrelin function and autophagy underlying OA pathogenesis warrants further investigations.

We profiled the differentially expressed circRNAs using a cellular OA model in rat chondrocytes in the present study. We further identified circPan3 targeting miR-667-5p and investigated their roles in regulating ghrelin synthesis, chondrocyte autophagy, and cartilage homeostasis-related markers.

## Materials and Methods

### Chondrocyte Isolation and Treatment

Knee joint tissues were collected from 6-day-old Sprague Dawley rats. Primary rat articular chondrocytes were isolated from the knee joint tissues as described previously, with minor modifications (Gosset et al., 2008; Zhou et al., 2018). Briefly, bilateral knee joint cartilage was rinsed twice with PBS and then digested with trypsin at 37°C for 30 min in a thermal incubator supplied with 5% CO_2_. Then the tissue fragments were incubated overnight in a 0.1–0.2% collagenase II solution at 37°C (Jiang et al., 2016). After filtering and centrifuging at 400 × *g* for 10 min, rat articular chondrocytes were seeded and cultured in GIBCO DMEM/F12 (1:1) medium (No.: C11330500BT), supplemented with 10% fetal bovine plasma (FBS; Gibco) and 1% penicillin and streptomycin (Sigma Aldrich). Cells grew on 60 mm culture plates at 37°C in a humidified incubator supplied with 5% CO_2_. Cells were passaged when they reached 80–90% confluent. Chondrocytes within P3 generation were used in this study.

Rat chondrocytes were treated with recombinant rat IL-1β (cat. No. I2393; Sigma Aldrich; 10 ng/ml) for 12 or 24 h to establish an *in vitro* OA cellular model as previously described (Huang et al., 2019). Chondrocytes cultured under normal conditions without IL-1β were set as normal control (NC) group.

### Quantitative Real-Time Polymerase Chain Reaction and Reverse Transcription Polymerase Chain Reaction

The relative expressions of mRNAs, miRNAs, and circRNAs in chondrocytes and rat articular cartilage were measured by quantitative real-time polymerase chain reaction (RT-qPCR), as described previously (Zhou et al., 2019). Briefly, total RNA was extracted from cells or tissues using the Invitrogen^TM^ TRIzol^TM^ Reagent (cat. No. 15596026; Thermo Fishier Scientific). Then, complementary DNA (cDNA) library was synthesized using the TransScript Reverse Transcriptase (MMLV) (#AT101-02; TransGene Biotech, Beijing, China) according to the manufacturer’s instructions. The expression levels of mRNAs, miRNAs, and circRNAs were finally determined by RT-qPCR using the SYBR Premix Ex Taq II (#RR820A; Takara Bio, Japan) according to the manufacturer’s instructions. GAPDH was used as an internal reference for the quantification of mRNAs and circRNAs (Zhou et al., 2019). U6 was used as an internal reference for quantifying miRNAs. To validate the circular properties of the predicted circPan3, regular reverse transcription polymerase chain reaction (RT–PCR) was carried out using cDNA or genomic DNA (gDNA) as the template, combined with divergent and opposite-directed primer pairs to determine circRNA and linear DNA, respectively. The primers are listed in Table 1.

**TABLE 1:** Primers applied for RT-qPCR method.

**Primer ID**	**Primer sequences (5′-3′)**	**Product length (bp)**
Col2a1-F	GGCCAGGATGCCCGAAAATTA	153
Col2a1-R	ACCCCTCTCTCCCTTGTCAC	
Acan-F	TTGATGAGTGCCTCTCAAGCC	84
Acan-R	TCGGAAGGCATAAGCATGTGA	
Ghrelin-F	GAGCTCAGTACCAGCAGCAT	91
Ghrelin-R	TACTTGTTAGCTGGCGCCTC	
circwdr33-F	TGCCCAGTGTTTGTTGTGAG	97
circwdr33-R	TGGAACCTCGGCATATGGAA	
circsox6-F	GGACGGAACAAGAGGAAGAA	116
circsox6-R	TGCCCTCTTCCTTTTCCCTT	
circscaper-F	TATCCAGGGGCGTGAACTTT	144
circscaper-R	CCATGTGCCTTCGAATGCTT	
circZfpm1-F	AGTGGCCCAGGTTCCCTC	134
circZfpm1-R	TCTTCTCTGGGTGGACTTGG	
miR-667-5p-F	AACAATCGGTGCTGGTGGA	68
miR-667-5p-RT	GTCGTATCCAGTGCAGGGTCCGAGG TATTCGCACTGGATACGACGTGCTC	
miR-667-5p-R	GTCGTATCCAGTGCAGGGT	
GAPDH-F	GCAAGAGAGAGGCCCTCAG	74
GAPDH-R	TGTGAGGGAGATGCTCAGTG	
U6-F	CTCGCTTCGGCAGCACA	94
U6-R	AACGCTTCACGAATTTGCGT	
circpan3-LF	TACCGGGCTTCCAAATCCAT	155
circpan3-LR	GTGTATGGCTTGGCACTGTC	
circpan3-CF	CTCCAGGCTGAGTAACGTGT	199
circpan3-CR	TACCGGGCTTCCAAATCCAT	

### Western Blotting

Total proteins from cultured chondrocytes or cartilage tissues were extracted using radioimmunoprecipitation assay (RIPA) lysis buffer (Beyotime, China). The protein concentration was measured using the bicinchoninic acid (BCA) protein assay (Beyotime, China). After boiling at 100°C for 5 min, protein samples were subjected to separation on SDS–PAGE and blotted onto PVDF membranes. Subsequently, the PVDF membranes were blocked with a 5% skimmed milk solution for 2 h at room temperature, followed by incubation with primary antibodies overnight at 4°C. Then, membranes were incubated with corresponding secondary antibodies for 1 h at room temperature. Bands were developed using the Pierce^TM^ ECL Western Blotting Substrate (#32106; Thermo Fishier Scientific). GAPDH was used as the internal standard. The antibodies used in this study were anti-Col2a1 (#ab34712; Abcam, Cambridge, United Kingdom; 1:4000), anti-aggrecan (#ab36861; Abcam; 1:5000), anti-ghrelin (#31865; CST, Danvers, United States; 1:4000), anti-LC3 (#4108; CST; 1:5000), anti-beclin1 (#ab210498; Abcam; 1:4000), MMP13 (ab39012, Abcam; 1:5000), ADAMTS5(ab41037, Abcam;1:250), and anti-GAPDH (#ab181602; Abcam; 1:5000) antibodies.

### Profiles of circRNAs and mRNAs

The deep-sequencing method was employed to determine the differentially expressed circRNAs and mRNAs in IL-1 β-treated chondrocytes compared with control chondrocytes, as described previously (Yan et al., 2017). Briefly, total RNA samples were extracted from chondrocytes after the indicated treatments. The RNA integrity was evaluated using an Agilent 2100 Bioanalyzer. The fragment size of RNA was determined by agarose gel electrophoresis. The rRNA components were removed from the RNA samples using the Qiagen RiboMinus Eukaryote Kit, according to the manufacturer’s instructions. Subsequently, the RNA library for sequencing was established from the mixed RNA samples (from three independent experiments) using the NEBNext Ultra II RNA Library Prep Kit (#E7770S; NEB, United States) according to the protocol provided by the manufacturer. Finally, the constructed RNA library was sequenced using a Hiseq 2000 instrument (Illumina, United States). Clean reads from sequencing were aligned with the rat reference genomic database using the Bowtie2 software, as described previously (Langmead and Salzberg, 2012). Then we applied the back-splice algorithm for the selection of read junctions. Subsequently, the function prediction and annotation of circRNAs were carried out using the CIRI software, which was designed for *de novo* circRNA identification (Gao et al., 2015). The mapped back-splicing junction reads per million mapped reads (RPM) values were then calculated by normalizing to the total read numbers. RPM was used for assessing the circRNA relative expression levels. Significant differences in circRNA expression between the two groups were determined using *P*-values ≤ 0.05 and Log2Ratios ≥ 1 as the thresholds. The differentially expressed mRNA profiles between the two groups were also generated by RNA sequencing, according to a previous report (Bauer et al., 2010), using *P*-values ≤ 0.05 and Log2Ratio ≥ 0.59 to define significantly differentially expressed mRNAs.

### Bioinformatics Analysis

The hierarchical clustering of differentially expressed circRNAs and mRNAs in both groups’ chondrocytes was presented as heap maps created using the pheatmap (version 1.0.10). The functional categorization of differentially expressed mRNAs was performed using Visualization and Integrated Discovery (DAVID) database, based on gene ontology (GO) biological processes, molecular functions, and subcellular components. The signaling pathway enrichment of differentially expressed mRNAs was analyzed using the Kyoto Encyclopedia of Genes and Genomes (KEGG) database. The receiver operating characteristic (ROC) curve analysis was carried out using the SPSS 20.0 software.

### Animal Model and Histological Evaluation

Animals were maintained under standard conditions (12-h light/12-h dark cycle at 20–24°C and 50–55% humidity) for one week before surgery. In total, 20 male 12-week-old Sprague Dawley rats were randomly divided into the OA model group (*n* = 10) and the control group (*n* = 10). OA model in rats was fabricated as described previously (Appleton et al., 2007). Briefly, all animals were anesthetized with ketamine (100 mg/ml), xylazine (20 mg/ml), and acepromazine (10 mg/ml) dissolved in saline (0.9% solution) at a dose of 100 μl/100 gm body weight. The OA group rats were exposed the medial aspect of the right knee joint capsule and performed anterior cruciate ligament transection and partial medial meniscectomy. The rats in the control group were performed a sham surgery: a similar operation in the same joint positions without anterior cruciate ligament transection and partial medial meniscectomy. Then trisbrissen antibiotic (Schering Canada, Pointe Claire, QC, Canada) was administrated for three days to avoid infection. And rats in both groups were forced mobilization for 4 weeks: 30 min, three times per week. Four weeks after the operations, the rats were sacrificed. Their right knee joints were surgically collected for histological evaluations via H&E and safranin O/fast green staining (Song et al., 2018). The Osteoarthritis Research Society International (OARSI) cartilage OA grading system was employed to determine the extent of cartilage degeneration (Glasson et al., 2010). A total of three different areas from the medial tibial plateau were randomly selected to calculate and evaluate the OARSI score. The sections were examined blindly by two researchers, respectively, and the scores were averaged to minimize observer bias. At the same time, blood samples were collected from the abdominal aorta and stored in a vacutainer. The blood samples were centrifuged at 1075 *g* for 20 min to collect plasma for biochemical analysis. The ghrelin, IL-1β, and IL-6 contents in the plasma were measured using the Rat GHRL ELISA Kit (#E-EL-R0842c; Elabscience, Wuhan, China), the Rat IL-1βELISA Kit (#20210628CN; Meimian, Jiangsu, China), and the Rat IL-6 ELISA Kit (#E-EL-R0015c; Elabscience, Wuhan, China) according to the manufacturer’s instructions. The Experimental Animal Ethics Committee of Forevergen Medical Laboratory Animal Center approved the animal operations mentioned above.

### Cell Transfections

In order to overexpress circPan3 in rat chondrocytes, a recombinant pcDNA3.1-circPan3 vector was constructed. CircPan3-coding sequences were amplified via RT–PCR and inserted into pcDNA3.1. We used miR-667-5p mimics to overexpress miR-667-5p in rat chondrocytes. MiR-667-5p mimics (forward, 5′–CGGUGCUGGUGGAGCAGUGAGCAC–3′; and reverse, 5′–GUGCUCACUGCUCCACCAGCACCG–3′) and mimics negative control (mimics NC) (forward, 5′–UUCUCCGAACGUGUCACGU–3′; and reverse, 5′–ACGUGACACGUUCGGAGAA–3′) sequences were synthesized at the GenePharma Company (Shanghai, China). When reaching 70–80% fusion in a 6-well plate, cells were transfected with the above plasmid vector or mimics and their respective control using lipofectamine 3000 reagent (Thermo Fishier Scientific) according to the manufacturer’s instructions.

### Dual-Luciferase Reporter Assay

The dual luciferase reporter assay was used to reveal the interaction between circPan3, miR-667-5p, and *ghrelin* gene 3′-UTR (untranslated regions). The assays were carried out using the Nano-Glo Dual-Luciferase Reporter (NanoDLR) Assay (Promega) according to the manufacturer’s instructions. The wild-type (wt) circPan3 sequences (circPan3 wt1: 5′–TTCCCGGAATGGATGGAGGTGCTTTAACTGACACAAGCC TCACAGATTCCTATTTCAGCACCAGCTTCATTGGAGTG AATGGATTTGGAAGCCCGGTAGAAACAAAGTATCCCT TGATGC–3′; and circPan3 wt2: 5′–AGACACCAAATCCTACT GCAAGCGAATTTATACCTAAAGGAGGATCAACCTCCAGG CTGAGTAACGTGTCCCAGTCAAATATGTCTGCCTTCTC TCAAGTTTTCTCTCACCCATCCATGGGAAGTCCTGCTAC TGCTGGATTAGCACCAG–3′), the corresponding mutant type (mut) sequences (circPan3 mut1: 5′–TTCCCGGAATGGA TGGAGGTGCTTTAACTGACACAAGCGAGTGACATAGGT TTTTGTCGTGGAGCTTCATTGGAGTGAATGGATTTGGAA GCCCGGTAGAAACAAAGTATCCCTTGATGC–3′; and circPan3 mut2: 5′–AGACACCAAATCCTACTGCAAGC GAATTTATACCTAAAGGAGGATCAACCTCCAGGCTGAGT AACGTGTCCCAGTCAAATATGTCTGCCTTCTCTCAAGTT TTCTCTCACCCATCCATGGGAAGTCCTCGATGACGAGGT TTTCGTGGAG–3′), ghrelin-3′-UTR-wt1 sequence (5′–CCAC TGACAGGACTGGTCCCTGTACTTTCCTCCTAAGCAAGAA CTCACATCCAGCTTCTGCCTCCTCTGCAACTCCCAGCAC TCTCCTGCTGACTTACAAATAAATGTTCAAGCTGT–3′), and ghrelin-3′-UTR-mut1 sequence (5′–CCACTGACAGGACT GGTCCCTGTACTTTCCTCCTAAGCAAGAACTCACATCCA GCTTCTGCGACGTGACGAAGTCGGTCGTGTCTCCTGCT GACTTACAAATAAATGTTCAAGCTGT–3′) were synthesized and ligated with the pmirGLO Vector (Promega). The above sequences were inserted between *Sac*I and *Sal*I sites, respectively. These recombinant plasmids were transfected into rat chondrocytes combined with miR-667-5p mimics or its negative control sequences as described above. The luciferase activity was finally determined using a GloMax-20/20 luminometer. According to the manufacturer’s instruction (Promega), when miR-667-5p binds to its target inserted, the firefly luciferase expressed will be reduced.

### Confocal Laser Scanning Microscope

HBAD-mcherry-EGFP-LC3 adenovirus (MOI = 50) was used to infect rat articular chondrocytes for 24 h according to the manufacturer’s instructions (Hanbio, Shanghai, China). Then cells were divided into four groups to transfect using lipofectamine 3000 reagent: pcDNA3.1 CircRNA Mini Vector + miR-NC (pcDNA3.1 + miR-NC), circPan3 + miR-NC (circPan3 + miR-NC), pcDNA3.1 CircRNA Mini Vector + rno-miR-667-5p (pcDNA3.1 + miR-667-5p), and circPan3 + rno-miR-667-5p (circPan3 + miR-667-5p) group. After 24 h, the autophagosomes were photographed using Confocal Laser Scanning Microscope (CLSM).

### Statistical Analysis

Quantitative data were presented as the mean ± standard deviation (SD). The SPSS 20.0 software was used for the statistical analysis. Unpaired Student’s *t*-test was used for the comparison between two groups. One-way analysis of variance (ANOVA) was used for the comparison between three groups and above. A *P*-value of <0.05 indicates a statistical significance.

## Results

### Expressions of Ghrelin and Autophagy-Related Markers Decreased in the Cellular Osteoarthritis Model

**FIGURE 1:**
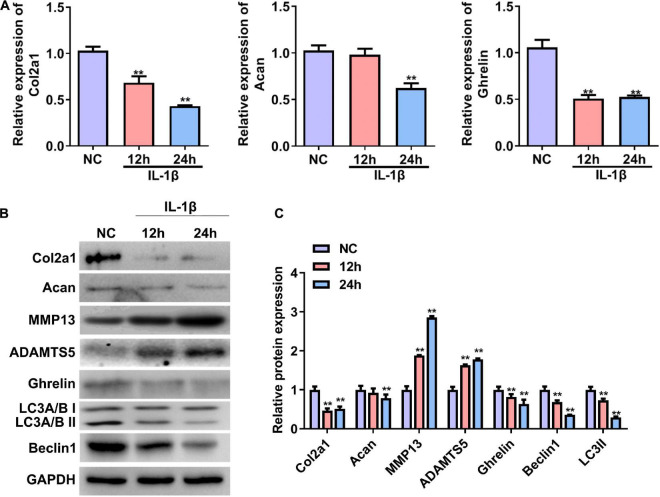
Expression of the ghrelin, autophagy-related markers, and cartilage matrix markers in rat chondrocytes stimulated with IL-1β. **(A)** Relative mRNA levels of the Col2a1, Acan, and ghrelin in rat chondrocytes after IL-1β stimulation. RT–qPCR was used to determine mRNA levels. **(B)** Western blotting results of the Col2a1, Acan, ghrelin, MMP13, ADAMTS5, beclin 1, LC3, and GAPDH in chondrocytes stimulated with IL-1β. **(C)** Quantification of the proteins shown in **(B)**. RT–qPCR, quantitative real-time polymerase chain reaction; NC, normal control; IL-1β, interleukin-1β; Col2a1, collagen 2a1; Acan, aggrecan; MMP13, matrix metallopeptidase 13; ADAMTS5, ADAM metallopeptidase with thrombospondin type 1 motif 5; GAPDH, glyceraldehyde-3-phosphate dehydrogenase. **P < 0.01 versus NC, *n* = 3.

We first established a cellular OA model by treating primary rat chondrocytes with IL-1β. The RT–qPCR and western blotting results showed that the mRNA and protein expression of Col2a1 and Acan were significantly reduced in rat chondrocytes treated with IL-1β for 24 h (Figures 1A–C). In contrast, the protein expressions of MMP13 and ADAMTS5 were significantly increased in chondrocytes induced by IL-1β (OA group) compared with chondrocytes without IL-1β stimulation (NC, Figures 1B,C). These results demonstrate that an *in vitro* cellular OA model was successfully established. The mRNA and protein expression of ghrelin were less in the IL-1β-induced chondrocytes than that in the NC group (Figures 1A–C). In the meantime, beclin 1 and LC3-II expressions were decreased in chondrocytes insulted by IL-1β (Figures 1B,C).

### Differentially Expressed circRNAs Profile in IL-1β-Induced Rat Chondrocytes

**FIGURE 2:**
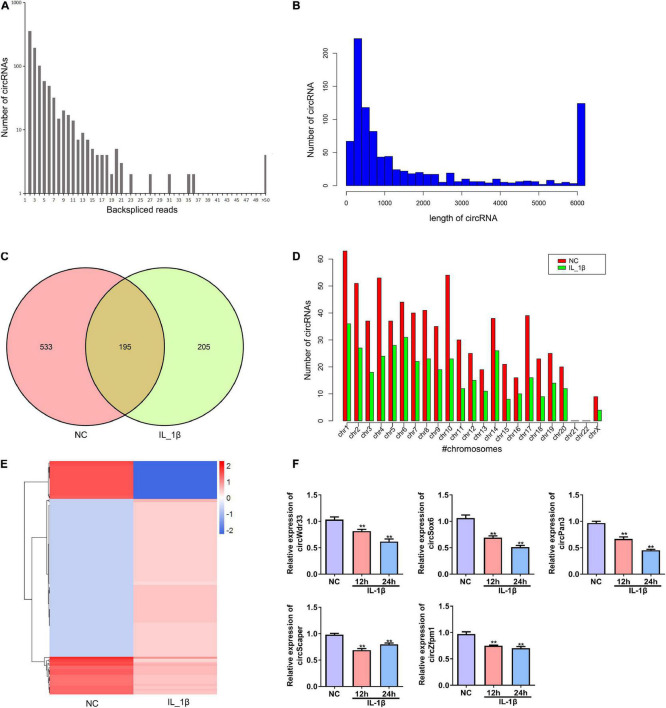
Profile of differentially expressed circRNAs in rat chondrocytes stimulated with IL-1β. **(A)** The number of back-spliced reads of the circRNAs identified in chondrocytes by deep sequencing. The number of back-spliced reads and circRNAs are indicated in the *X* and *Y* axis, respectively. **(B)** Length distribution of identified circRNAs. **(C)** Venn diagram showing the number of circRNAs identified in the NC and IL-1β-induced chondrocytes. **(D)** Chromosomes distribution of the circRNAs. The circRNAs identified in the NC and IL-1β-induced chondrocytes are indicated by red and green bars, respectively. **(E)** Hierarchical clustering of differentially expressed circRNAs. CircRNAs between two groups with a *P*-value < 0.05 and a Log_2_ ratio ≥ 1 were considered differentially expressed. **(F)** Relative expression of five representative differentially expressed circRNAs in IL-1β-induced rat chondrocytes and the corresponding NC cells. The expression of circWdr33, circSox6, circPan3, circScaper, and circZfpm1 in chondrocytes was determined by RT–qPCR. RT–qPCR, quantitative real-time polymerase chain reaction; NC, normal control; IL-1β, interleukin-1β; **P < 0.01 versus NC, *n* = 3.

Next, we performed deep RNA sequencing to reveal the profile of differentially expressed circRNAs between OA chondrocytes and control chondrocytes. Most circRNAs were identified in rat chondrocytes with 2 to 36 back-spliced reads (Figure 2A). The lengths of the circRNAs covered an extensive range, with the largest number at a size near 200 bp (Figure 2B). In total, 933 circRNAs were identified from rat chondrocytes in both groups: 728 and 400 circRNAs in the negative control and IL-1β-treated chondrocytes, respectively. Among these circRNAs, 195 circRNAs were identified in both groups (Figure 2C and Supplementary Table 1). According to their encoded gene location, these circRNAs were widely scattered on almost all chromosomes, except for chromosomes 21 and 22 (Figure 2D). The majority of circRNAs were located on chromosome 1 (Figure 2D). We identified a total of 130 differentially expressed circRNAs, including 107 upregulated and 23 downregulated circRNAs in the OA group compared with the NC group (Figure 2E and Supplementary Table 2). Based on the ceRNA mechanism, circRNAs that potentially target and regulate ghrelin were predicted. We selected the top five circRNAs for validation by RT-PCR: IL-1β stimulation decreased the expressions of circWdr33, circSox6, circPan3, circScaper, and circZfpm1 in chondrocytes (Figure 2F). The expression trends of circSOX6, circPan3, and circZfpm1 were consistent with the sequencing results (Figure 2F). Thus, these three circRNAs were further studied in the subsequent experiments.

### The Profile of Differentially Expressed mRNAs in Rat Chondrocytes Stimulated With IL-1β

**FIGURE 3:**
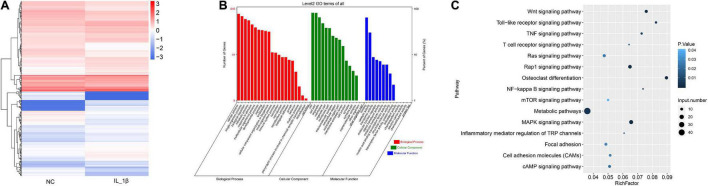
The profile of differentially expressed mRNAs in rat chondrocytes stimulated with IL-1β. **(A)** Hierarchical clustering of differentially expressed mRNAs between IL-1β-induced rat chondrocytes and normal control chondrocytes. The chondrocyte mRNAs between two groups exhibiting a *P*-value < 0.05 and a Log_2_ ratio ≥ 0.59 were considered as differentially expressed genes. **(B)** Functional categorization of differentially expressed mRNAs. Genes were categorized separately based on GO biological processes, cellular components, and molecular functions. **(C)** KEGG analysis results of enrichment of differentially expressed mRNAs. NC, normal control; IL-1β, interleukin-1β; GO, gene ontology; KEGG, kyoto encyclopedia of genes and genomes.

We further profiled the differentially expressed mRNAs in rat chondrocytes stimulated by IL-1β. A total of 732 differentially expressed mRNAs were identified in IL-1β-induced rat chondrocytes, including 410 upregulated and 322 downregulated genes (Figure 3A and Supplementary Table 3). The GO functional categorization revealed that these differentially expressed genes were mainly enriched in various biological processes, such as metabolism, responses to stimulus, development, signaling, localization, immune system, locomotion, reproduction, and biological adhesion (Figure 3B). Moreover, these differentially expressed mRNAs were distributed into various subcellular components and had distinct molecular functions (Figure 3B). These differentially expressed genes were mainly enriched in several signaling pathways, including osteoclast differentiation, toll-like receptor signaling, Wnt (Wingless and INT-1) signaling, TNF signaling, and NF-κB signaling pathways (Figure 3C).

### Decreased Expression of Ghrelin, circPan3, and Autophagy Biomarkers and in the Rat Osteoarthritis Model

**FIGURE 4:**
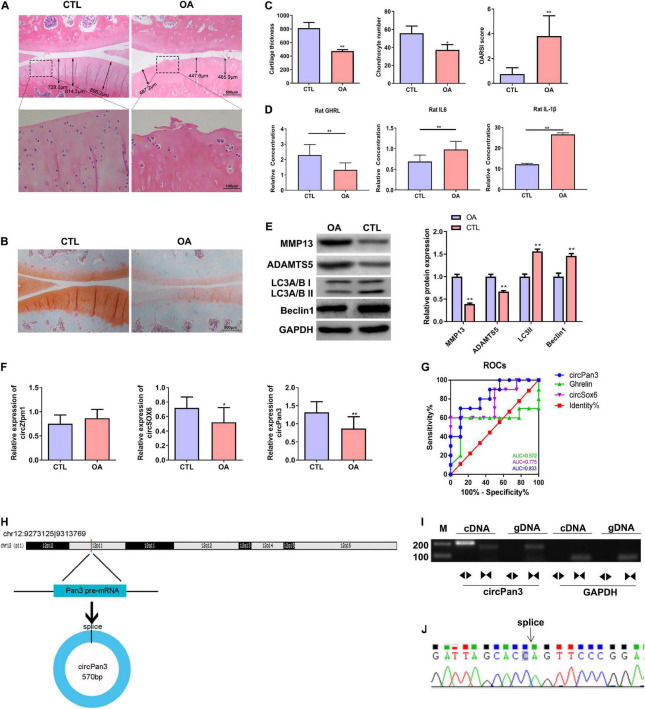
Decreased circPan3 expression, ghrelin synthesis, and autophagy in the rat OA model. **(A)** Histological alterations of knee joint cartilage tissues in OA and CTL rats. The rat cartilage tissues were subjected to H&E staining. Rats that underwent the sham operation were used as the control group (CTL). **(B)** Safranin O/fast green staining of knee joint cartilage tissues from both groups. **(C)** The analysis results of cartilage thickness, chondrocyte density, and OARSI scores in knee joint tissues from both groups. **(D)** Plasma GHRL, IL-6, and IL-1β levels in both groups. The ELISA method was used to determine the levels of these factors. **(E)** Western blotting results of MMP13, ADAMTS5, beclin 1, and LC3 in the cartilage tissues of rats from both groups. **(F)** The expressions of circZfpm1, circSox6, and circPan3 in the knee joint cartilage tissues of rats in the OA model group. The relative expression levels of circRNAs were measured by RT–qPCR. **(G)** ROC curve analysis of circPan3, circSox6, and ghrelin as potential biomarkers of OA pathogenesis. **(H)** Bioinformatics predicted that circPan3 was formed from the back-splicing of *Pan3* encoded pre-mRNA, which is located at chromosome 12. **(I)** Validation of the formation of circPan3 in rat chondrocytes. RT–PCR was carried out using divergent and opposite-directed primers and cDNA and gDNA as the templates. **(J)** Confirmation of the splice joint site of circPan3 in rat chondrocytes was assessed by DNA sanger sequencing. The joint site was indicated by an arrow. CTL, control; OA, osteoarthritis; OARSI, Osteoarthritis Research Society International; GHRL, ghrelin; IL-6, interleukin-6; IL-1β, interleukin-1β; MMP13, matrix metallopeptidase 13; ADAMTS5, ADAM metallopeptidase with thrombospondin type 1 motif 5; GAPDH, glyceraldehyde-3-phosphate dehydrogenase; RT–qPCR, quantitative real-time polymerase chain reaction; RT–PCR, reverse transcription polymerase chain reaction; ELISA, enzyme linked immuneSorbent assay; ROC, receiver operating characteristic; cDNA, complementary DNA; gDNA, genomic DNA. *P < 0.05; **P < 0.01 versus CTL, *n* = 3.

We established a rat OA model as described in the Material and Methods section. H&E staining showed that the knee joint cartilage tissues of OA rats exhibited a rough surface, heterogenous staining, a faint tidal line, decreased cartilage thickness, an irregular chondrocyte lining, and a reduced chondrocyte density compared with those of the control group (Figures 4A,C). In line with H&E staining results, safranin O/fast green staining showed damaged cartilage tissues, decreased cartilage thickness, and an uneven distribution of matrix components in the OA group compared with the control group (Figures 4B,C). Moreover, OARSI scores in the OA group were remarkably higher than those in the control group (Figure 4C). These biochemical and histological evaluations demonstrated that a rat OA model was successfully established.

Next, the ELISA assay showed that the GHRL content was significantly decreased while IL-6 and IL-1β contents were significantly upregulated in the plasma in the OA group compared with the control group (Figure 4D). IL-1β is considered as one of the most critical cytokines involved in the development of OA (Jenei-Lanzl et al., 2019). The elevation of IL-1β in the plasma of OA rats indicates that chondrocytes stimulated with IL-1β in this study could serve as an *in vitro* OA model (Jenei-Lanzl et al., 2019). In addition, western blotting results showed that the protein levels of beclin 1 and LC3-II were markedly decreased while MMP13 and ADAMTS5 protein expressions were significantly increased in the OA rats compared with the control group, indicating that the autophagy process was inhibited in the OA rat cartilage tissues (Figure 4E).

We further determined the expressions of circSOX6, circPan3, and circZfpm1 in the rat OA model. We found that the expression of circSox6 and circPan3 but not circZfpm1 was significantly decreased in the cartilage tissues of rats in the OA group compared with the control group (Figure 4F). A subsequent ROC curve analysis showed that the AUC (area under the curve) of circPan3 for OA pathogenesis was remarkably higher than that of circSox6 and ghrelin (Figure 4G, *P* < 0.05), suggesting that circPan3 is a candidate OA biomarker. Next, bioinformatics analysis indicated that circPan3 was formed by the back-splicing of the pre-mRNA sequences in *Pan3* gene located on rat chromosome 12 (Figure 4H). To validate this predicted result, we examined the junction sites of circPan3 by RT–PCR, results showed products from divergent primers targeting circPan3 only amplified from cDNA of rat chondrocytes (Figure 4I). And subsequent DNA Sanger sequencing for this products further confirm the junction sites of circPan3 (Figure 4J). These results demonstrate that the circPan3 expression in rat chondrocytes is reliable.

The above results suggested that upregulated IL-1β and decreased expressions of circPan3, ghrelin, and insufficient autophagy are potentially associated with OA pathogenesis.

### CircPan3 Promoted Ghrelin Synthesis and Chondrocyte Autophagy and Protected Against Osteoarthritis Development by Directly Targeting miR-667-5p

**FIGURE 5:**
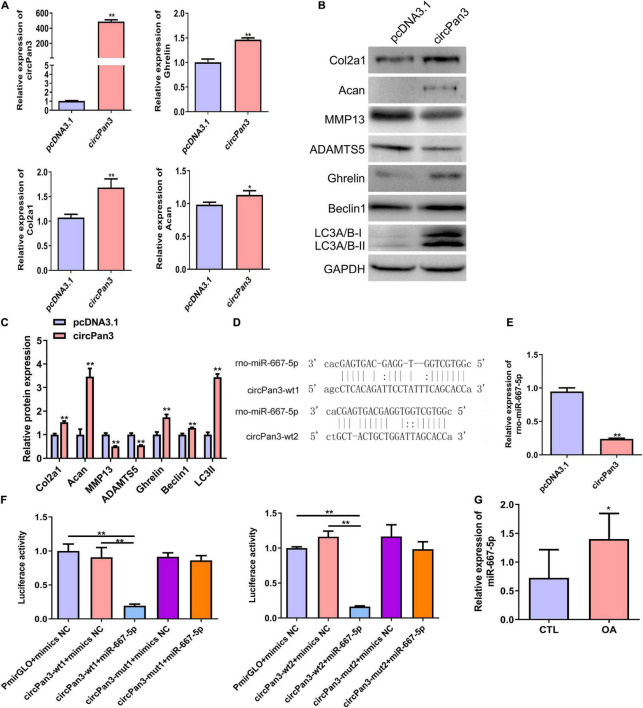
CircPan3 directly target miR-667-5p and promoted ghrelin synthesis and chondrocyte autophagy. **(A)** Relative expression levels of circPan3, ghrelin, Col2a1, and Acan in rat chondrocytes with or without overexpressing circPan3. Expression levels of circRNAs and the indicated mRNAs were assessed by RT–qPCR. **(B)** Western blotting results of Col2a1, Acan, MMP13, ADAMTS5, ghrelin, beclin 1, and LC3 in rat chondrocytes with or without overexpressing circPan3. **(C)** Quantitative analysis of western blotting in **(B)**. **(D)** Two binding sites of circPan3 to miR-667-5p were predicted by bioinformatics analysis. **(E)** Relative expression levels of miR-667-5p in rat chondrocytes with or without overexpressing circPan3. The miR-667-5p expression was assessed by RT–qPCR. **(F)** Validation of the direct binding between circPan3 and miR-667-5p in rat chondrocytes by dual luciferase reporter assay. **(G)** The miR-667-5p expression in cartilage tissues of rats from CTL and OA group was determined by RT-qPCR. RT–qPCR, quantitative real-time polymerase chain reaction; Col2a1, collagen 2a1; Acan, aggrecan; MMP13, matrix metallopeptidase 13; ADAMTS5, ADAM metallopeptidase with thrombospondin type 1 motif 5; GAPDH, glyceraldehyde-3-phosphate dehydrogenase; wt, wild type; mut, mutant; CTL, control; OA, osteoarthritis. *P < 0.05; **P < 0.01, *n* = 3.

To explore the role of circPan3 in OA pathogenesis, we overexpressed circPan3 in cultured rat chondrocytes. Overexpression of circPan3 resulted in significantly increased ghrelin, Col2a1, and Acan mRNA expressions in rat chondrocytes compared with the pcDNA3.1 group, which was transfected with the empty plasmid (Figure 5A). Western blotting results showed that the protein levels of the ghrelin, beclin 1, LC3-II, Col2a1, and Acan were significantly elevated in circPan3-overexpressed chondrocytes compared with the pcDNA3.1 group (Figures 5B,C). In contrast, the protein expressions of MMP13 and ADAMTS5 significantly decreased in circPan3-overexpressed chondrocytes (Figures 5B,C). Thus, circPan3 promoted cartilage matrix anabolism and inhibited catabolism to protect against OA development. Bioinformatics analysis indicated that circPan3 might bind to miR-667-5p at two different sites (Figure 5D). And miR-667-5p expression in rat chondrocytes was significantly reduced by overexpressing circPan3 (Figure 5E). Thus, we carried out a dual-luciferase reporter assay to determine whether circoPan3 can target miR-667-5p directly. We observed that miR-667-5p transfection caused a significant decrease in luciferase activities in chondrocytes expressing the wild-type circPan3 sequences but not in those expressing the mutant circPan3 sequences (Figure 5F). These results demonstrate that circPan3 binds to miR-667-5p directly at two distinct sites. Furthermore, miR-667-5p expression significantly increased in OA cartilage tissues than those in CTL group (Figure 5G). The above results presented that circPan3 targeting miR-667-5p directly regulates ghrelin synthesis and chondrocyte autophagy during OA development.

### miR-667-5p Suppressed Chondrocyte Autophagy and Promoted Osteoarthritis Progression by Targeting Ghrelin

**FIGURE 6:**
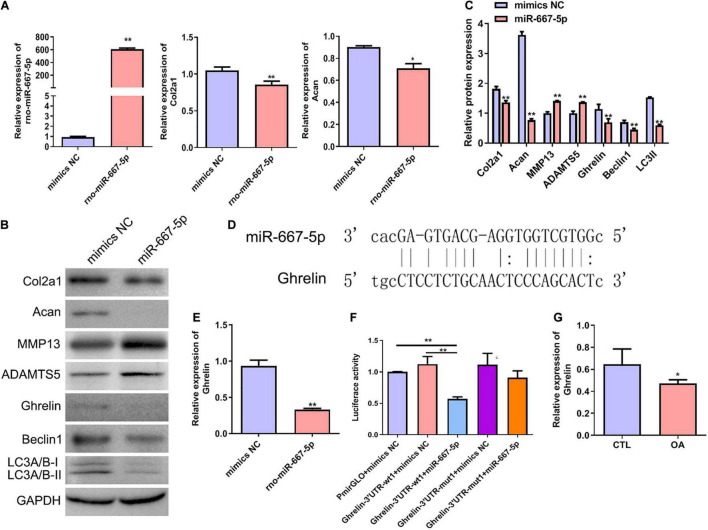
miR-667-5p inhibited autophagy and promoted OA progression likely by targeting *ghrelin* gene in chondrocytes. **(A)** Expression levels of miR-667-5p, Col2a1, and Acan in rat chondrocytes transfected with mimics NC or miR-667-5p mimics. The relative levels of miRNAs and mRNAs were determine using RT–qPCR. **(B)** Western blotting results of Col2a1, Acan, MMP13, ADAMTS5, ghrelin, beclin 1, and LC3 in rat chondrocytes transfected with mimics NC or miR-667-5p mimics. **(C)** The quantitative analysis results of western blotting shown in **(B)**. **(D)** Binding site between miR-667-5p and the *ghrelin* gene 3′-UTR sequences was predicted by bioinformatics analysis. **(E)** RT-qPCR results of *ghrelin* gene expression in rat chondrocytes transfected with mimics NC or miR-667-5p mimics. **(F)** Validation of the direct binding of miR-667-5p to the 3′-UTR sequences in *ghrelin* gene in rat chondrocytes via dual luciferase reporter assay. **(G)** The *ghrelin* expression in cartilage tissues of rats from CTL and OA group was determined by RT-qPCR. mimics NC, mimics negative control; RT–qPCR, quantitative real-time polymerase chain reaction; Col2a1, collagen 2a1; Acan, aggrecan; MMP13, matrix metallopeptidase 13; ADAMTS5, ADAM metallopeptidase with thrombospondin type 1 motif 5; GAPDH, glyceraldehyde-3-phosphate dehydrogenase; UTR, untranslated regions; wt, wild type; mut, mutant; CTL, control; OA, osteoarthritis. *P < 0.05; **P < 0.01, *n* = 3.

To further investigate the function of miR-667-5p in OA and its downstream mechanism, we transfected rat chondrocytes with miR-667-5p mimics to overexpress miR-667-5p (Figure 6A). In miR-667-5p-overexpressed rat chondrocytes, the mRNA levels of Col2a1 and Acan genes were significantly decreased compared with the mimics NC group (Figure 6A). Western blotting results also showed that the protein levels of Col2a1, Acan, ghrelin, beclin 1, and LC3-II were remarkably decreased while the protein expressions of MMP13 and ADAMTS5 were significantly increased in the miR-667-5p overexpressed group compared with the mimics NC group (Figures 6B,C). Therefore, miR-667-5p prevented cartilage matrix anabolism and accelerated catabolism leading to OA development. The bioinformatics analysis predicted that miR-667-5p binds to the 3′-UTR sequences of the ghrelin gene (Figure 6D). And overexpression of miR-667-5p significantly reduced ghrelin mRNA expression in chondrocytes (Figure 6E). Next, we used a dual-luciferase reporter assay to determine the interaction between ghrelin and miR-667-5p. The results showed that miR-667-5p mimics induced a significant decrease in luciferase activity in rat chondrocytes expressing wild-type 3′-UTR sequences of *ghrelin* gene but not in chondrocytes expressing mutant 3′-UTR sequences of *ghrelin* gene (Figure 6F). Moreover, ghrelin mRNA expression was reduced in OA cartilage tissues compared with those in CTL group (Figure 6G). These findings exhibited that miR-667-5p regulates chondrocyte autophagy and OA progression by targeting *ghrelin*.

### CircPan3 Protects Against Osteoarthritis Development via Targeting miR-667-5p

**FIGURE 7:**
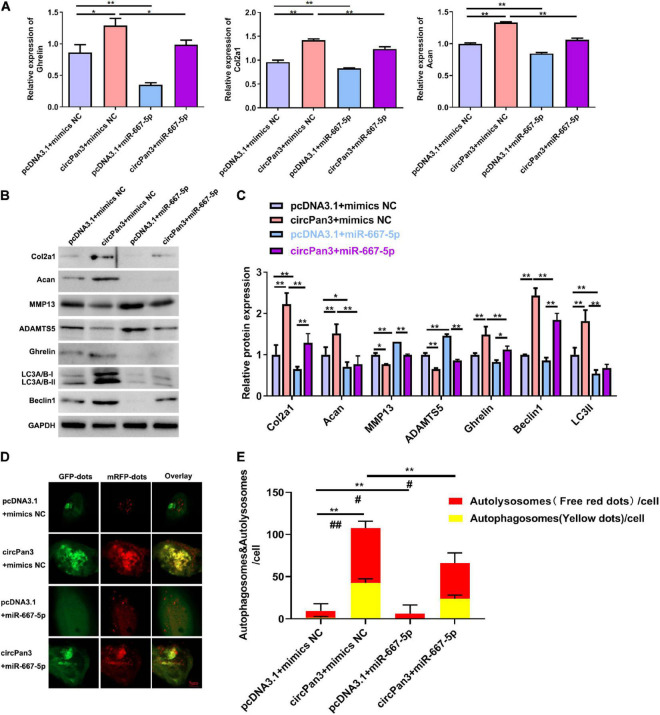
The effects of miR-667-5p on the circPan3-regulated ghrelin synthesis, chondrocyte autophagy, and OA progression. **(A)** RT-qPCR results of ghrelin, Col2a1, and Acan gene expressions in rat chondrocytes treated as indicated. **(B)** Western blotting results of Col2a1, ghrelin, Acan, MMP13, ADAMTS5, beclin 1, and LC3 in rat chondrocytes treated as indicated. **(C)** Quantitative analysis results of western blotting in **(B)**. **(D)** Autophagy in rat chondrocytes was identified by confocal laser scanning microscope. Yellow fluorescence represents autophagosomes and red fluorescence represents autolysosomes, respectively. **(E)** The average number of yellow dots (autophagosomes) and red dots (autolysosomes) per cell in each group was analyzed. mimics NC, mimics negative control; RT–qPCR, quantitative real-time polymerase chain reaction; Col2a1, collagen 2a1; Acan, aggrecan; MMP13, matrix metallopeptidase 13; ADAMTS5, ADAM metallopeptidase with thrombospondin type 1 motif 5; GAPDH, glyceraldehyde-3-phosphate dehydrogenase; *P < 0.05; **P < 0.01; #*P* < 0.05; ##*P* < 0.01 versus the respective control group, *n* = 3.

Finally, we investigated whether circPan3 served as miR-667-5p sponge to protect against OA development. We observed that miR-667-5p mimics reversed the regulative effects of circPan3 on the mRNA expressions of ghrelin, Col2a1, and Acan in chondrocytes (Figure 7A). The western blotting results of ghrelin, Col2a1, and Acan were consistently similar to RT-qPCR results (Figures 7B,C). In addition, western blotting also showed that miR-667-5p mimics also blunted the effects of circPan3 on the protein expressions of MMP13, ADAMTS5, beclin 1, and LC3-II in chondrocytes (Figures 7B,C). We further determined the effects of the interaction between circPan3 and miR-667-5p on autophagy in chondrocytes. As shown in Figures 7D,E, autophagosome numbers were prominently elevated in circPan3 + mimics NC, and this elevation was weakened by circPan3 + miR-667-5p. This trend is consistent with the results in Figures 7B,C. Together, these results demonstrated that circPan3 promoted ghrelin synthesis and chondrocyte autophagy to protect against OA development via targeting miR-667-5p.

## Discussion

Osteoarthritis is a common joint disease characterized by the dysregulation of chondrocytes and the decrease in extracellular matrix components, its’ pathogenesis is closely associated with chondrocyte autophagy, and ghrelin prevented articular cartilage matrix destruction in human chondrocytes (Zhang et al., 2015; Zou et al., 2016; Liu et al., 2018; Qu et al., 2018; Yu and Zhao, 2019). However, the molecular mechanisms underlying the regulation of the ghrelin and autophagy changes in OA chondrocytes remain poorly understood. This study showed that ghrelin synthesis and autophagy were decreased in both cellular and rat OA models. Through deep sequencing and further validation, we showed for the first time that the circRNA circPan3 was significantly decreased in both *in vivo* and *in vitro* OA models. We further revealed that circPan3 promoted ghrelin synthesis and chondrocyte autophagy, thus protecting against OA development via directly binding to miR-667-5p and thus suppressing miR-667-5p expression in chondrocytes. Moreover, our results showed that miR-667-5p directly bound to the 3′-UTR sequence of the *ghrelin* gene.

Autophagy is characterized by the lysosome-mediated degradation and recycling of cellular components, which is implicated in cell death (Anding and Baehrecke, 2015; Ha and Kim, 2016). The activation of autophagy is usually accompanied by elevated expression of LC3-II and beclin 1, which are used widely as autophagy biomarkers (Russo et al., 2011; Zhong et al., 2016). A recent study showed that autophagy was reduced in OA cartilage (Feng et al., 2020). Activation of autophagy could inhibit apoptosis and degradation in chondrocytes (Caramés et al., 2012; Shapiro et al., 2014). Thus, activation of autophagy is considered beneficial for the survival of chondrocytes in the early stage of OA (Duan et al., 2020). In the present study, we showed the suppression of the protein expressions of beclin 1 and LC3-II in both *in vivo* and *in vitro* OA models. The ghrelin system regulated the induction of autophagy in several cell types, such as retinal neuronal and vascular cells (Xu et al., 2017; Zhu et al., 2017). Decreased synovial fluid ghrelin levels are related to disease severity in patients with primary OA. The synovial fluid ghrelin levels were increased following laser therapy. Ghrelin may play a protective role in knee OA (Zou et al., 2017). In this study, we observed that ghrelin mRNA and proteins expressions significantly decreased in both *in vitro* and *in vivo* OA models. Our results also showed that the ghrelin content increased in OA cartilage tissues. These findings suggest that the existence of a regulatory relationship between ghrelin and autophagy likely plays a critical role in OA pathogenesis (Sun et al., 2020; Wang et al., 2021).

To explore the molecular mechanisms underlying the modulation of ghrelin and chondrocyte autophagy in OA development, we profiled the differentially expressed circRNAs and mRNAs between rat chondrocytes stimulated with or without IL-1β using deep sequencing. A large number of differentially expressed circRNAs were identified during OA development in chondrocytes. The differentially expressed circRNAs might play essential pathogenic roles in OA through targeting their downstream signaling pathways. Among these circRNAs, we showed that the expression of circPan3 was significantly suppressed in IL-1β-induced rat chondrocytes and cartilage tissues of OA rats. Our subsequent analysis revealed that circPan3 promoted ghrelin synthesis and chondrocyte autophagy but suppressed OA development, as evidenced by the upregulation of Col2a1 and Acan and the decrease of MMP13 and ADAMTS5 in chondrocytes. CircPan3 was formed by the back-splicing of the pre-mRNA encoding the PAN3 protein, which formed a heterodimer with the PAN2 protein and was involved in miRNA-mediated mRNA degradation (Christie et al., 2013; Jonas et al., 2014). Previous reports showed that other circRNAs derived from the human *Pan3* gene regulated drug resistance in acute myeloid leukemia through modulating autophagy and apoptosis (Shang et al., 2019a,b), indicating that circPan3 might exert similar biological functions in chondrocytes. CircPan3 might also regulate autophagy in other biological and pathogenic processes, which warrants further investigation. Moreover, a large number of differentially expressed genes associated with essential biological processes and signaling pathways were identified in this study, providing a basis for future analyses of circRNA-related gene expression in OA pathogenesis.

The major biological functions of circRNAs are mediated by their capacities of sponging miRNA or work as competing endogenous RNAs (ceRNAs) (Han et al., 2017; Zhang et al., 2019). CircSERPINE2 protected against OA by targeting miR-1271 and ETS-related genes (Shen et al., 2019). Circular RNA Atp9b regulated the progression of OA by targeting miR-138-5p (Zhou et al., 2018). In this study, we further found that circPan3 targeted miR-667-5p, and miR-667-5p expression increased in OA cartilage tissues. Moreover, we found that miR-667-5p targeted the 3′-UTR sequences of the *ghrelin* gene, resulting in decreased ghrelin mRNA and protein expressions in chondrocytes. We further verified that circPan3 regulating OA development was mediated by miR-667-5p: miR-667-5p overexpression blunted the protective effects of circPan3 against OA chondrocytes and inhibited autophagy activation induced by circPan3 overexpression. Previous studies have shown that the ghrelin system is closely associated with autophagy under various biological and pathogenic conditions (Delporte, 2013; Mani and Zigman, 2017; Xu et al., 2017; Zhu et al., 2017). Our findings suggest that circPan3/miR-667-5p axis might regulate autophagy via ghrelin-mediated signaling in chondrocytes during OA development.

Further studies are required to confirm the effects of knockdown of circPan3/miR-667-5p on OA development. Furthermore, our study should further verify the effect of circPan3/miR-667-5p in human OA cartilage tissues and human cells. We will determine the functions of circPan3/miR-667-5p/ghrelin in human knee cartilage tissues of normal individuals and OA patients through both overexpression and knockdown approaches. Besides, we did not determine the effects of ghrelin on chondrocyte autophagy. Ghrelin is a potential regulator of autophagy in metabolic, cardiac or neuronal disorders (Ezquerro et al., 2017). Our findings demonstrate that circPan3 suppresses miR-667-5p and thus enhancing autophagy in chondrocytes, indicating that ghrelin as a target of miR-667-5p might mediate the effects of circPan3/miR-667-5p axis on chondrocyte autophagy.

In summary, this study uncovered the profiles of differentially expressed circRNAs and mRNAs in chondrocytes during OA development. Our findings demonstrate that circPan3 promotes ghrelin synthesis and autophagy activation in chondrocytes by sponging miR-667-5p that targets *ghrelin* gene, providing experimental evidence that circPan3/miR-667-5p/ghrelin signaling axis might serve as potential targets of drug development for OA treatment.

## Data Availability Statement

The data presented in the study are deposited in the GEO dataset, accession number GSE186993 (https://www.ncbi.nlm.nih.gov/geo/query/acc.cgi?acc=GSE186993).

## Ethics Statement

The animal study was reviewed and approved by the Experimental Animal Ethics Committee of Forevergen Medical Laboratory Animal Center.

## Author Contributions

L-XY and GL conceived and designed the study and critically revised the manuscript. JZ, Z-ZZ, and QL performed the experiments, analyzed the data, and drafted the manuscript. Q-JL and J-ML participated in study design, study implementation, and manuscript revision. All authors read and approved the final manuscript.

## Conflict of Interest

The authors declare that the research was conducted in the absence of any commercial or financial relationships that could be construed as a potential conflict of interest.

## Publisher’s Note

All claims expressed in this article are solely those of the authors and do not necessarily represent those of their affiliated organizations, or those of the publisher, the editors and the reviewers. Any product that may be evaluated in this article, or claim that may be made by its manufacturer, is not guaranteed or endorsed by the publisher.
